# Effect of maternal skin-to-skin contact on decolonization of Methicillin-Oxacillin-Resistant Staphylococcus in neonatal intensive care units: a randomized controlled trial

**DOI:** 10.1186/s12884-015-0496-1

**Published:** 2015-03-19

**Authors:** Fernando Lamy Filho, Sílvia Helena Cavalcante de Sousa, Isolina Januária Sousa Freitas, Zeni Carvalho Lamy, Vanda Maria Ferreira Simões, Antônio Augusto Moura da Silva, Marco Antônio Barbieri

**Affiliations:** Departamento de Saúde Pública, Universidade Federal do Maranhão, Rua Barão de Itapary, 155, Centro, São Luís, Maranhão Brazil; Departamento de Puericultura e Pediatria (7° andar HCRP), Faculdade de Medicina de Ribeirão Preto – USP, Universidade de São Paulo, Av. Bandeirantes, 3900 – Campus USP, Ribeirão Preto, Brazil

**Keywords:** Skin-to-skin contact, Decolonization, MRSA/MRSE

## Abstract

**Background:**

Decolonization with topical antibiotics is necessary to control outbreaks of multidrug-resistant bacterial infection in the Neonatal Intensive Care Unit (NICU), but can trigger bacterial resistance. The objective of this study was to determine whether skin-to-skin contact of newborns colonized with Methicillin-Oxacillin Resistant *Staphylococcus aureus* or Methicillin-Oxacillin-Resistant Coagulase-Negative *Staphylococcus aureus* (MRSA/MRSE) with their mothers could be an effective alternative to promote bacterial decolonization of newborns’ nostrils.

**Methods:**

We performed a randomized clinical trial with 102 newborns admitted to the NICU in three hospitals in São Luís, Brazil. Inclusion criteria were birth weight of 1300 to 1800 g, more than 4 days of hospitalization, newborns with positive nostril cultures for MRSA and/or multidrug-resistant coagulase-negative Staphylococcus and mothers not colonized by these bacteria. We used a random number algorithm for randomization. Allocation was performed using sealed opaque envelopes. Skin-to-skin contact was given twice a day for 60 minutes for seven consecutive days. The control group received routine care without skin-to-skin contact. There was no masking of newborn’s mothers or researchers but the individuals who carried out bacterial cultures and assessed results were kept blind to group allocation. The primary outcome was colonization status of newborns’ nostrils after 7 days of intervention. The directional hypothesis was that more newborns who receive skin-to-skin holding 2 hours/day for 7 days than newborns who receive normal care will be decolonized.

**Results:**

Decolonization of MRSA/MRSE was greater in the intervention group (Risk Ratio = 2.27; 95% CI 1.27-4.07, p-value = 0.003). Number Needed to Treat (NNT) was 4.0 (95% CI 2.2 – 9.4). After adjustment for the possible confounding effects of small for gestational age birth, antibiotic use, need for resuscitation, sex and cesarean delivery, skin-to-skin contact remained strongly associated with decolonization of newborns’ nostrils from MRSA/MRSE bacteria (p = 0.007). There was no need to interrupt the trial for safety reasons.

**Conclusion:**

Skin-to-skin contact might be an effective and safe method for promoting decolonization of newborns’ nostrils colonized by MRSA/MRSE.

**Trial Registration:**

The study was registered with ClinicalTrials.gov (NCT01498133, November 21, 2011).

## Background

Staphylococcus resistant to methicillin-oxacillin is one of the most frequent pathogens colonizing newborns (NB) admitted to Neonatal Intensive Care Units (NICU) [1]. They are identified as being primarily responsible for outbreaks of nosocomial infection especially in situations of overcrowding and understaffing [2,3]. Mupirocin promotes decolonization of these bacteria, but does not prevent outbreaks of infection and can trigger bacterial resistance [4-6].

Studies suggest that the presence of nonpathogenic bacteria can inhibit MRSA growth. Uehara et al. [7] showed that colonization by MRSA could be inhibited by the presence of methicillin non-resistant bacteria (Streptococcus viridans group) in the oral cavity of newborns admitted to neonatal units. Shimizu et al. [8] also showed the same effect on preterm infants admitted to the NICU of Nagano Children's Hospital.

Other studies have indicated the possibility of transmission of MRSA from mother to newborn through skin-to-skin contact. In 2003, Kawada et al. postulated that transmission of MRSA from mother to infant could occur through breastfeeding [9]. Sakaki et al. [10] found an association between skin-to-skin contact and newborn MRSA infection.

Several studies have also shown that certain bacteria of the normal flora of human skin and mucous membranes have the ability to take the place of multiresistant bacteria that are already installed, through a competitive mechanism termed bacterial interference [11,12]. This mechanism has been used to promote healing of infections by multiresistant bacteria mainly in the fields of urology and otorhinolaryngology [13,14]. It is also possible that this mechanism could be responsible for the ability of the Kangaroo Mother Care to reduce infection rates of newborns undergoing this method, as demonstrated by Lawn et al. in 2010 [15] and Conde-Agudelo, et al., 2011, in a Cochrane database meta-analysis [16].

Kangaroo mother care (KMC) is an effective and safe alternative to conventional neonatal care in low birthweight (LBW) infants that was found to reduce mortality at discharge or 40–41 weeks’ postmenstrual age and at latest follow up, severe infection/sepsis, nosocomial infection/sepsis, hypothermia, severe illness, lower respiratory tract disease, and length of hospital stay. The major component of KMC is skin-to-skin contact (SSC) between a mother and her newborn. Recently Lawn et al. [15] demonstrated that Kangaroo mother care is effective in preventing neonatal deaths due to preterm birth complications [17].

As literature points to an association between KMC and reduction of infections in preterm newborns, we tested the hypothesis of whether skin-to-skin contact between newborns colonized by MRSA/MRSE and their mothers is associated with decolonization of newborns’ nostrils.

## Methods

### Trial design and settings

We performed a controlled parallel randomized and single-blind clinical trial, conducted at the NICU of three public maternity hospitals in São Luís, northeastern Brazil: Hospital of the Federal University of Maranhão (HUMI), Marly Sarney Maternity Hospital (MMS) and Benedito Leite Maternity Hospital (MBL).

### Sample

A target sample of 100 patients (including possible losses to follow-up) was calculated considering a 30% difference in the percentage of decolonization between the intervention and control groups, with 80% power and 5% probability of type I error, assuming that percentage of decolonization in the control group is 20% and setting the ratio between groups at 1:1.

### Participants

Eligible subjects were singleton neonates, born at the three institutions of the study, weighing 1300 to 1800 g and clinically stable. They had been hospitalized for more than 4 days and their nostrils were colonized by *Staphylococcus aureus* or coagulase-negative Staphylococcus resistant to methicillin-oxacillin. Mothers were not colonized in their nostrils by these bacteria and did not present skin diseases.

#### Included infants and hospital participation

A total of 247 (21 from HUMI; 180 from MMS; 46 from MBL) dyads (mother and newborn) were assessed for eligibility from April 2008 to December 2010. The different number of patients assessed for eligibility in the three study hospitals was due to differences in size and number of hospitalizations in these units. Moreover, in the HUMI unit, data collection had to be discontinued because the skin-to-skin position was instituted as routine care, making randomization impossible.

A total of 102 dyads were found to be eligible for the study.

#### Excluded infants

**Figure 1:**
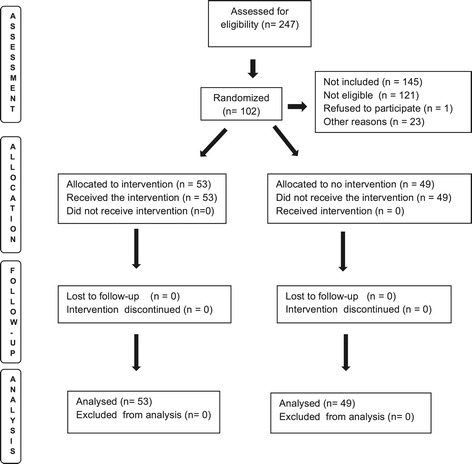
**Selection, allocation, intervention, monitoring and analysis of the patients enrolled in the study.** São Luís, Brazil, 2008–2010.

The remaining 145 newborns were not included in the study, 121 because they were not colonized with MRSA/MRSE and/or because their mothers were colonized with MRSA/MRSE at their first nostrils’ culture. One mother refused to participate and 23 did not participate for other reasons (Figure 1).

We did not include infants below 1300 g because they were often subjected to routine umbilical catheterization. Infants over 1800 g were excluded because they remained, in general, less than four days in the NICU. Those who underwent surgery for congenital problems, ostomy and urethral catheter drainage were not included as well.

#### Allocation

For allocation of participants, a computer-generated random number list was used. The allocation sequence was concealed by using sealed opaque black envelopes. After identification of each eligible dyad the chief researcher in the presence of the mother in the NICU opened an envelope. Groups were then formed (intervention group, n = 53; control n = 49).

Mothers and researchers were aware of group allocation (intervention or control), whereas the individuals who carried out the bacterial cultures and assessed the results were kept blind to the allocation.

### Interventions

Mothers in the study group were instructed to have skin-to-skin contact with their newborns in the NICU twice a day (morning and evening) for 60 minutes, for seven days (including weekends). Adherence to the intervention was verified daily and recorded on sheets. Skin-to-skin contact consisted of placing the infant wearing only a diaper in prone decubitus, upright against his mother's chest, between the breasts. The infant was restrained in position by a strap that tied him/her to his/her mother [18] and was covered with the mother’s clothes. NICU had its temperature maintained at 26 degrees Celsius.

All mothers underwent a routine hand washing procedure before entering the NICU. They did not have their chests scrubbed before skin-to-skin contact. The mother sat in a chair positioned by the side of the infants’ bed. Standing nurses transferred the babies to sitting mothers. A team member who accompanied the intervention monitored infant temperature, heart rate and oxygen saturation to ensure babies’ safety [19,20]. Both groups received routine nursing care such as nutrition, hygiene, bathing and diapering, organization of parents’ visit, breastfeeding and administration of drugs. Mothers were encouraged to touch, breast feed her baby and get him/her as soon as possible in her lap, under staff supervision. Fathers did not hold infants in skin-to-skin contact.

All mothers in the intervention group successfully completed 60 minutes of skin-to-skin contact for just one hour twice a day.

### Outcomes

The primary endpoint for testing the efficacy of intervention was colonization status of newborns’ nostrils after 7 days of intervention (decolonization of the infants’ nostril from multi-drug resistant Staphylococcus). Birth weight (measured at birth using digital scales with 5 gram precision), gestational age (according the the last menstrual date), type of delivery (vaginal/cesarean section), sex (male/female), birth weight for gestational age (classified according to Alexander’s curve) [21], 5th min Apgar score and need for resuscitation (at delivery room) and antibiotic use (from birth to the end of data collection) were compared between groups.

### Interim analysis and protocol of interruption

No interim analysis was performed. There was no need to interrupt the trial for safety reasons.

### Data collection

The material for the first bacterial culture was collected at baseline from both mothers and their newborns by a nasal swab performed on the fourth, fifth or sixth day of hospitalization, by a lab technician using a cotton swab soaked in sterile saline solution that was introduced into the nasal cavity of newborns and their mothers. The results of the culture from the first collection of nasal swabs determined the eligibility of the dyads for randomization.

Decolonization was checked by a second swab collection seven days after the beginning of the intervention. The second culture was collected only from infants. No other site of culture collection was considered in addition to the nostrils. Collected materials were placed in Stuart transport medium and sent to the laboratory for seeding in 5% Agar sheep blood and Brain Heart Infusion (BHI) for 24 to 48 h at 35 °C. Cultures were considered to be positive when Staphylococcus was isolated by the catalase, coagulase and VitekbioMerieux® automated method. Antimicrobial susceptibility testing was performed by Kirby Bauer disc diffusion, following recommendations from the CLSI/2008. For the samples considered to be “methicillin-oxacillin resistant” the E-test was used for confirmation of sensitivity to vancomycin.

All newborns who remained colonized after the second nostril culture, performed 7 days after randomization, were decolonized according to the recommendations of the Hospital Infection Control Committee of each unit at the end of the 7 days.

### Statistical analysis

Following CONSORT guidelines, we did not perform a statistical test comparing differences in baseline characteristics because of randomization.

For the analysis of primary outcome, we first applied the Mantel-Haenszel chi-square test for two proportions. In a second analysis we fitted a generalized linear model for the binomial family with a log link to control for possible confounding effects of small for gestational age birth, antibiotic use, need for resuscitation, sex and cesarean delivery. These variables were chosen based on the magnitude of differences in their distributions between the intervention and the control group. A p-value of less than 0.05 was considered statistically significant. All tests were two-tailed. To evaluate the clinical relevance of the outcome we calculated the Number Needed to Treat (NNT). Intention to treat analysis was not performed because there were no losses to follow-up.

### Ethical considerations

The study was approved by the Ethics Research Committee of the University Hospital, Federal University of Maranhão, Brazil, under No. 33104-1504/07 on behalf of all three participating hospitals. Each hospital’s director gave institutional permission for the study. All newborns’ mothers read a Plain Language Statement, written in plain, simple language, explaining the purpose, methods, demands, risks and potential benefits of the research and signed a written informed consent form. This trial was registered with ClinicalTrials.gov under number NCT01498133.

## Results

Mother-newborn dyads were recruited from April, 2008 through December, 2010. The flowchart in Figure 1 shows selection, allocation, intervention, monitoring and analysis of the patients enrolled in the study.

We assessed 247 newborns for eligibility. The eligible dyads (102) were distributed as follows: 25 at the University Hospital Unit, 175 at the Marly Sarney Maternity Hospital and 47 at the Benedito Leite Maternity Hospital. Among the 102 randomized patients, 83 infants had MRSA in the first culture and 19 had MRSE. Prevalence of MRSA/MRSE was 66.9%.

No participants were lost to treatment or follow-up after assignment to study groups and none of the newborns had changes in vital signs that required team interventions or stopping the procedure.

Baseline variables for the study groups are displayed in Table 1. The variables cesarean section, small for gestational age, need for resuscitation in the delivery room and antibiotics use were unbalanced between groups.

**Table 1:** ** Perinatal characteristics of preterm infants**

	**Study group**	**Control group**
	**(N = 53)**	**(N = 49)**
Mean ± SD*		
Birth weight	1524.05 ± 157.37	1509.08 ± 172.84
Gestational age	32.0 ± 2.4	32.2 ± 2.3
5th minute Apgar score	8.64 ± 1.0	8.62 ± 0.73
Percentage (%)		
Male sex	49.1	50.9
Born by cesarean delivery	60.4	51,0
Small for gestational age birth	62.3	37.7
Needed resuscitation in the delivery room	43.1	34,7
Antibiotics use	52.8	76.1

*SD = standard deviation.

São Luís, Brazil, 2008-2010 (n = 102).

### Outcomes and estimation

Decolonization rates were significantly different between groups, as illustrated in Table 2. Infants receiving skin-to-skin care were 2.35 times more likely to decolonize than the control group.

**Table 2:** **MRSA/MRSE decolonization in the infants’ nostrils in NICU in the intervention (skin-to-skin contact between mothers and newborns) and control groups**

**Groups**	**Decolonization**	**No decolonization**	RR*	95% CI**	p-value***
	**n (%)**	**n (%)**			
Skin-to-skin contact	28 (52.8 %)	25 (47.2 %)	2.35	1.32-4.20	0.002
Control	11 (22.4 %)	38 (77.6 %)			
Total	39 (38.2 %)	63 (61.8 %)			

São Luís, Brazil, 2008–2010.

*RR – risk ratio.

**CI – confidence interval.

***P-value estimated by the Mantel-Haenszel chi-square test.

Number Needed to Treat (NNT) was 4 (95% CI 2.2 – 9.4).

Even after adjustment for confounders skin-to-skin contact remained strongly associated with decolonization of newborns’ nostrils from MRSA/MRSE bacteria (Risk ratio = 2.30, 95% CI 1.30-4.06, p = 0.004) (Table 3).

**Table 3:** **Adjusted analysis of MRSA/MRSE decolonization of the nostrils of preterm infants admitted to the NICU (intervention vs. control group)**

**Variables**	Risk ratio*	95% CI**	**P-Value**
Small for gestational age birth	1.26	0.86-1.85	0.228
Antibiotic use	1.54	0.98-2.39	0.056
Skin-to-skin contact	2.30	1.30-4.06	0.004
Need for resuscitation	1.65	1.08-2.51	0.020
Born by cesarean delivery	0.81	0.57-1.15	0.245

São Luís, Brazil, 2008–2010 (n = 102).

*Estimated by a generalized linear model for the binomial family with a log link.

**CI – confidence interval.

It is worth noting that among those infants who decolonized from MRSA/MRSE, the same *genera* of bacteria that had grown in their mother’s baseline culture was identified in the infant’s second culture in 84.2% of cases (data not shown).

## Discussion

More than half of the newborns who received skin-to-skin holding intervention from their mothers who were not colonized with MRSA/MRSE were decolonized at the end of the seven days of treatment, but the mechanism for decolonization is unclear.

Several researchers have suggested that the presence of nonpathogenic flora [8] or strains of Streptococcus [7] inhibit growth of MRSA, possibly by acting on or interfering with some stage of the colonization process. A similar mechanism, replacement of newborn’s multi-resistant flora with mothers’ non-pathogenic flora, may also be implicated.

A possible explanation for this finding is the phenomenon of bacterial interference, through which mothers’ sensitive bacteria replace newborn’s MRSA/MRSE. This possibly occurs through changes in bacterial microenvironment that include competition for nutrients and production of antagonistic substances by mother’s bacteria such as bacteriocins. Recent works, especially in the area of urology and otorhinolaryngology, have shown that it is possible to induce exchange of multiresistant bacterial flora by introducing certain strains of antibiotic-sensitive bacteria [11-13]. It is possible that such mechanism could also explain the effect of skin-to-skin contact in reducing the incidence and severity of infection episodes in preterm infants, as observed in several studies [15,16].

In our study we found that children who decolonized from MRSA / MRSE had the same *genera* of bacteria of his mother's culture in 84.2% of cultures performed seven days after the beggining of the intervention. This increases the likelihood that replacement of infant’s multiresistant bacteria had occurred with their mother’s non-MRSA/MRSE bacteria.

The intensity of the effect of decolonization was demonstrated by the number needed to treat (NNT). Just four newborns had to undergo skin-to-skin contact for one decolonization to be observed, a potentially huge effect.

Decolonization of the control group could have been due to other factors present in the NICU. It is plausible that this fact occurred spontaneously or was influenced by other types of babies’ manipulation during routine care in the NICU. Kohler et al. describes spontaneous clearance rate (MRSA decolonization) of 22% [22]. Decolonization of 50% of infants who underwent skin-to-skin contact is of great importance since other methods of decolonization, such as the use of topical antibiotics and bathing with chlorhexidine, pose risk for premature babies, as shown by Nelson et al. in 2014 [4].

### Limitations

The impossibility of blinding mothers and researchers to the intervention could have led to differences in neonatal care between groups. However, the individuals who carried out the bacterial cultures and assessed the results were kept blind to the allocation. These differences are unlikely to have provoked changes in the results of bacterial cultures of nasal mucosa.

Colonization with non-pathogenic bacteria could also have been mediated through the individuals who moved the infant from the NICU bed to the skin-to-skin contact position with their mothers. However, contact time between these personnel and the newborns was short. In addition, all newborns had similar manipulation. Mothers and babies had not had any previous experience with skin-to-skin contact before the study, a fact that reduces the likelihood that mothers in the control group had performed skin-to-skin-contact during the study. Although data on breastfeeding have not been collected, breastfeeding routine was similar in both groups. Although colonization of the newborns’ nostrils in the intervention group could have occurred by any skin-to-skin contact, most skin-to-skin contact was provided by the kangaroo position.

No site of culture collection other than the nostrils was used in this work. While PCR for *mecA* is considered the gold standard assay for the detection of MRSA, the Vitek automated method used in our work is also reliable to detect MRSA, with sensitivity ranging from 90% to 99% and specificity close to 100% [23,24]. It is known that nasal swabs could be not so sensitive in assessing CA-MRSA colonization and that a negative test for nasal colonization does not rule out MRSA [25]. It is also known that colonization and/or culture yield can result in intermittently positive samples [26].

## Conclusions

Replacement of non-MRSA/MRSE bacteria from mothers to newborns through skin-to-skin contact could have occurred to explain a more than two-fold higher decolonization rate in the intervention group compared to the control group. The phenomenon of bacterial interference might be a possible mechanism explaining this finding.

The current methods of controlling bacterial outbreaks in the NICU are not effective in preventing endemic multiresistant Staphylococcus infection and can increase bacterial resistance [2,4,27,28]. The findings of this study might be a possible alternative to the decolonization of MRSA/MRSE from the infants’ nostrils because the procedure proved to be safe and effective and the number needed to decolonize one patient (NNT = 4.0) is superior to other methods of decolonization [28]. However, it is necessary to ensure that mothers eligible to practice skin-to-skin position with their babies are not carriers of MRSA/MRSE, since there is evidence in the literature that points to the possibility of transmission of this pathogen from mother to newborn [8,9].

Neonatal mortality by nosocomial infection remains one of the greatest challenges of public health [29-31]. Skin-to-skin contact between mothers and their newborns might be a safe and cost-effective strategy of biological control to promote decolonization of multiresistant bacteria and a possible reduction of nosocomial infections in the NICU.
